# A 10-year trend in cannabis potency (2013–2022) in different geographical regions of the United States of America

**DOI:** 10.3389/fpubh.2024.1442522

**Published:** 2024-10-03

**Authors:** Mahmoud A. ElSohly, Chandrani G. Majumdar, Suman Chandra, Mohammed M. Radwan

**Affiliations:** ^1^National Center for Natural Products Research, School of Pharmacy, University of Mississippi, University, MS, United States; ^2^Department of Pharmaceutics and Drug Delivery, School of Pharmacy, University of Mississippi, University, MS, United States

**Keywords:** cannabinoids, *Cannabis sativa*, gas chromatography with flame ionization detector, potency, CBD, Δ^9^-THC

## Abstract

**Background:**

The prevalence of cannabis as the most commonly used illicit substance in the United States and around the globe is well-documented. Studies have highlighted a noticeable uptrend in the potency of cannabis in the United states. This report examines the concentration of cannabinoids in illicit cannabis samples seized by the United States Drug Enforcement Administration (DEA) over the last 10 years (2013–2022).

**Methods:**

Samples received during the course of study (2013–2022) were categorized based on the geographical region where collected, as Western Region, Midwest Region, Northeast Region, South East Region, Southern Region as well as Alaska and Hawaii. These samples were processed for analysis using a validated gas chromatography with flame ionization detector method.

**Results:**

The data showed that the cannabinoids profile of all high Δ^9^-THC cannabis samples, regardless of the state or region from which the samples are seized or the state from which the sample is produced under a state medical marijuana program, is basically the same with the major cannabinoid being Δ^9^-THC (>10% for most samples) and all other cannabinoids with less than 0.5%, with the exception of CBG (<1%) and CBN (<1%).

**Conclusion:**

Overall, it appears the cannabinoids profile is controlled by the genetics of the plant and is not affected much by the geographical location in which the plants are cultivated.

## Introduction

As a plant, cannabis is valued for its euphorigenic and medicinal properties, as well as its industrial importance. In nature, it is found in various habitats at almost all elevations up to the foothills of the Himalayan mountains. The history of the cannabis plant goes back to over 10,000 years. It is one of the oldest sources of food and textile fiber. Hemp grown for fiber was introduced in Western Asia and Egypt and subsequently to Europe between 1000 and 2000 BCE. Over the last few years growing of cannabis (hemp) has become a major agricultural industry in numerous countries.

Cannabis has a long history of being used as a crude drug to treat epilepsy, tetanus, rheumatism, migraine, asthma, trigeminal neuralgia, fatigue, insomnia and many other ailments in the Middle East and Asia, with references as far back as the 6th century BCE. It was introduced in Western Europe as a medicine in the early 19th century. More recently its derivatives are being used in HIV/AIDS and multiple sclerosis. The plant contains a complex mixture of compounds. It produces a unique class of terpenophenolic compounds called cannabinoids. More than 550 constituents have been isolated from *Cannabis* so far, out of which 129 compounds are phytocannabinoids (1, 2). The concentration of cannabinoids in the dried inflorescence (leaves and buds) is considered to be the most objective measure to classify the plant according to the degree of psychoactivity since THC and other cannabinoids are produced in the glandular trichomes predominantly found in the buds and leaves of the plant. Based on the presence of the most abundant cannabinoids, THC and CBD, in its leaves and buds, *C. sativa* was divided into three distinct chemotypes namely, drug type (high THC chemotype), fiber type/hemp (high CBD chemotype), and intermediate chemotype (with balanced THC and CBD levels).

Although cannabis preparations have been used over millennia for their psychoactivity, as well as for their therapeutic properties, their chemistry and biology were not well known until the last few decades. Indeed, the major psychoactive cannabis constituent, Δ^9^-tetrahydrocannabinol (THC), was isolated in a pure form, and its structure was elucidated, only in the early 1960’s (3). This is in sharp contrast with the thorough knowledge for morphine and cocaine, which were isolated during the nineteenth century. However, since the 1960s, a large number of investigations have been devoted to the phytocannabinoids and more recently to the endocannabinoids fields. Today, the pharmacology and therapeutic efficacy of cannabis preparations and Δ^9^-tetrahydrocannabinol (Δ^9^-THC) have been extensively reviewed (4–8).

The other important cannabinoid of current interest is cannabidiol (CBD). There has been a significant interest in CBD over the last few years because of its reported activity as an antiepileptic agent, particularly its promise for the treatment of intractable pediatric epilepsy (9). Other than Δ^9^-THC and CBD, tetrahydrocannabivarin (THCV), cannabinol (CBN), cannabigerol (CBG), and cannabichromene (CBC) are major isolates. During the late nineteenth and early twentieth centuries, pharma-companies produced and marketed numerous cannabis containing remedies in Europe and the United States. Merck, Burroughs-Wellcome, Bristol-Meyers Squibb, Parke-Davis, and Eli Lilly marketed various cannabis extracts and tinctures. However, due to numerous concerns about illicit/recreational use and availability of synthetic alternatives, cannabis was dropped from the British Pharmacopeia in 1932 and from the United States Pharmacopeia in 1941. These concerns led to national and international laws restricting/regulating the medicinal use and research of cannabis.

Currently, cannabis is considered the most widely used illicit drug in the world and therefore highly regulated in the USA at the federal level. A growing number of studies have recently reported that higher potency cannabis preparations are associated with adverse health outcomes, including elevated symptoms of cannabis use disorder, increased emergency room admissions for cannabis problems, higher risk of developing psychosis, and increased risk of relapse to psychosis (10). Increases in cannabis potency and the associated health effects are especially significant among adolescents who are more vulnerable to cannabis harms (11). Therefore, it is very important to monitor the potency the confiscated biomass and cannabis products available on the market as a measure of what is actually being sold and consumed by the public.

Several recent reports from different parts of the world such as Norway, Turkey, The Netherlands, England, France and Italy (12) shows that the potency of cannabis and cannabis related products are constantly increasing. This is consistent with our previous studies in the USA reported in 2021, 2016, 2013, 2010, and 2000 (12–16). In a meta-analysis performed on 21 different studies worldwide, containing 75 observations on mean Δ^9^-THC levels in herbal cannabis samples, revealed a consistent increase in cannabis potency worldwide over time (17).

In our last cannabis potency study, we reported that cannabis potency in the United States increased from ~4% in 1996 to ~14% in the year 2019 (13). In this article, we report cannabis potency trends in the United States over the last 10 years (2013–2022). For the purpose of this study samples collected from all over USA were categorized by region of seizure, namely; Western Region, Midwest Region, Northeast Region, South East Region, Southern Region as well as Alaska and Hawaii, in order to determine if there is a regional difference in the type of cannabis used in the different regions of the country.

## Materials and methods

### Acquisition of Samples

**Figure 1:**
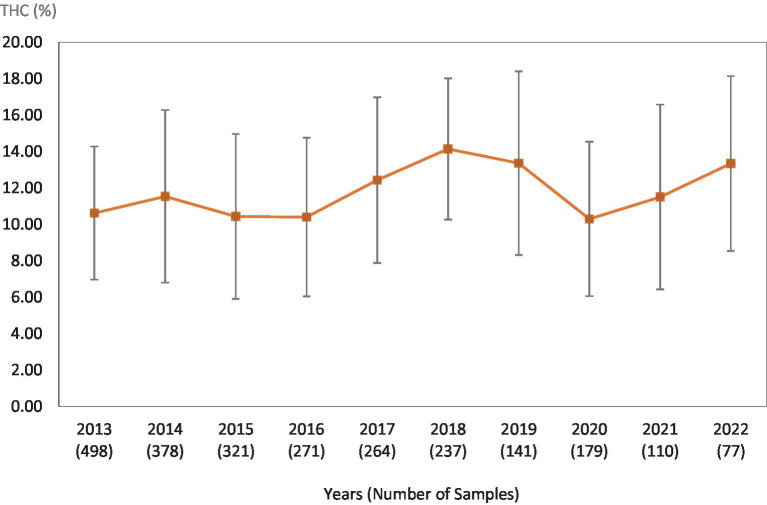
Average Δ^9^-THC (±SD) content of samples seized within the Western region of the USA over the last 10 years. SD is calculated from the mean values of the different states.

Under agreement between the National Institute on Drug Abuse (NIDA), and the Drug Enforcement Administration (DEA), confiscated cannabis samples are submitted by the DEA laboratories to our program for analysis. The DEA laboratories from which samples are received include, Special Testing Research Laboratory (STRL), Northeast Regional Laboratory (NRL), Mid-Atlantic Regional Laboratory (MARL), North Central Regional Laboratory (NCRL), South Central Regional Laboratory (SCRL), Southwest Regional Laboratory (SWRL), and Western Regional Laboratory (WRL). For the purpose of this report, samples collected from all over the USA were categorized, based on the geographical region where collected, as Western Region, Midwest Region, Northeast Region, South East Region, Southern Region as well as Alaska and Hawaii (Figure 1). All samples received are stored at room temperature (17 ± 4°C) until analyzed.

### Sample preparation for analysis

Samples were manicured by sieving (14 mesh) to remove the stems and seeds (if any), and duplicate 100 mg samples from each exhibit were weighed for analysis. Each of the two 100 mg samples was extracted with 3 mL of the internal standard solution [4-androstene-3,17-dione (IS), at 1 mg/mL in CHCl_3_/MeOH (1:9)] at room temperature for 1 h. The extract was then filtered and the filtrate was analyzed by gas chromatography with flame ionization detection (GC/FID), using our previously reported and validated method (18).

### GC-FID analysis

All samples were analyzed using a Varian 3380 gas chromatograph equipped with a Varian CP-8400 automatic liquid sampler, dual capillary injectors, and dual flame ionization detectors (GC/FID). The column was a 15 m × 0.25 mm DB-1, 0.25 μ film. Data were recorded with a Dell Optiplex GX1 computer with Microsoft Windows 98 and Varian Star (version 5.31) workstation software. Technical grade helium was used as the carrier gas. A high capacity oxygen trap was located in the helium line. Helium was used as the detector make-up gas. Hydrogen and compressed air were used as the combustion gases.

The method was used to quantitate seven major cannabinoids; namely, Δ^9^-tetrahydrocannabinol (Δ^9^-THC), cannabidiol (CBD), cannabinol (CBN), cannabichromen (CBC), Δ^8^-tetrahydrocannabinol (Δ^8^-THC), cannabigerol (CBG), and tetrahydrocannabivarin (THCV). Direct injection of cannabis extract into the GC results in decarboxylation of the cannabinoid acids, therefore, the concentration measured is for the total cannabinoids (free and acids). Quantitative values are based on peak area ratios relative to the area of the internal standard peak (4-androstene-3,17-dione) contained in the extraction solvent.

### Calculation of cannabinoid concentration

Quantitative values (% w/w) are computer generated based on the analyte/internal standard area ratio, with each cannabinoid having a response factor of 1.0. The concentration of each cannabinoid in the samples is calculated from the following equation:

$\begin{array}{l} \mathrm{Cannabinoid}\;\left(\%\right)=\\ \left\{\mathrm{Peak area}\;\left(\mathrm{cannabinoid}\right)/\mathrm{Peak area}\;\left(\mathrm{ISTD}\right)\right\}\times \\ \left\{\mathrm{Amount}\;\left(\mathrm{ISTD}\right)/\mathrm{Amount}\;\left(\mathrm{sample}\right)\right\}\times 100 \end{array}$

## Results and discussion

In previous studies, we have reported on the changes in cannabis potency (based on the Δ^9^-THC concentration) over time (12–16) in the United States. These reports provided the average Δ^9^-THC content of confiscated samples countrywide. With the legalization of cannabis in many states for medical or recreational use or both, and the fact that samples that are legal in one state cannot be moved to other states, brought the question whether cannabis biomass available in one state or a given region could be different from those in another region.

**Figure 2:**
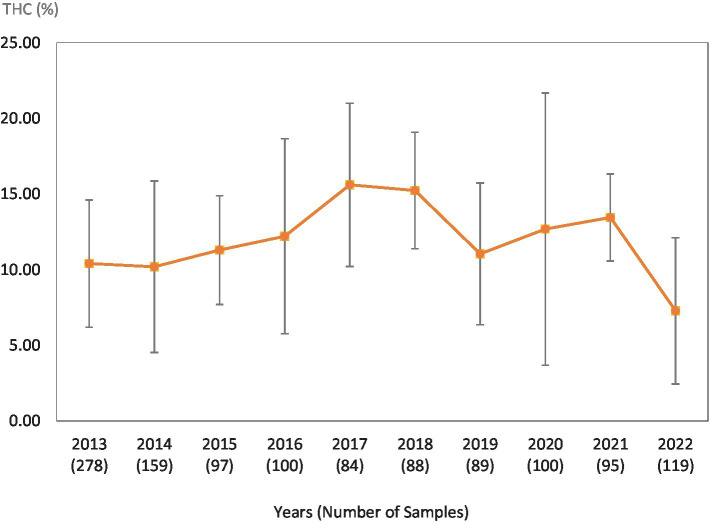
Average Δ^9^-THC (±SD) content of samples seized within the Midwestern region of the USA over the last 10 years. SD is calculated from the mean values of the different states.

**Figure 3:**
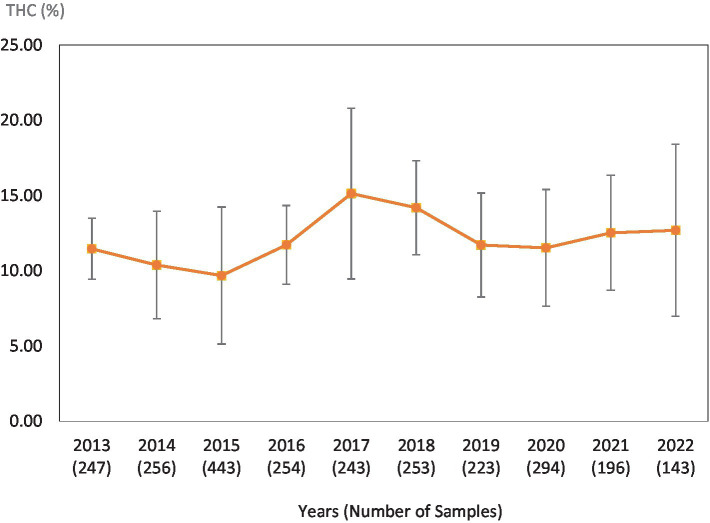
Average Δ^9^-THC (±SD) content of samples seized within the Southern region of the USA over the last 10 years. SD is calculated from the mean values of the different states.

**Figure 4:**
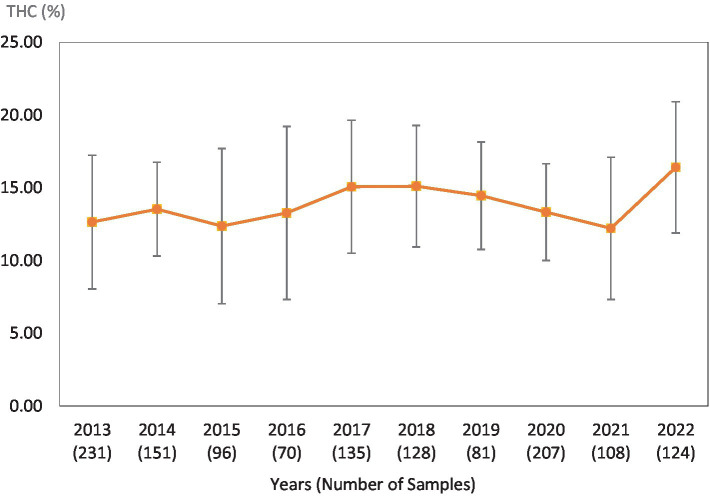
Average Δ^9^-THC (±SD) content of samples seized within the Northeastern region of the USA over the last 10 years. SD is calculated from the mean values of the different states.

**Figure 5:**
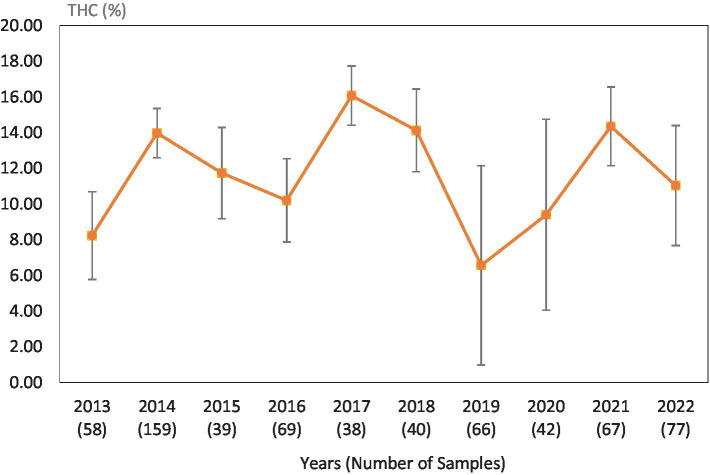
Average Δ^9^-THC (±SD) content of samples seized within the Southeastern region of the USA over the last 10 years. SD is calculated from the mean values of the different states.

**Figure 6:**
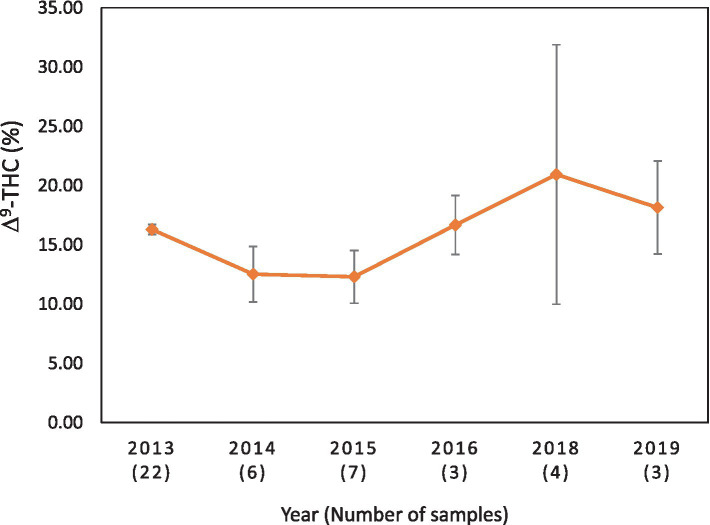
Average Δ^9^-THC (±SD) content of samples seized within the Alaska and Hawaii regions of the USA over the last 10 years. SD is calculated from the mean values of the different states. No sample was received from these states during the years 2017, 2020, 2021, 2022, and 2023.

This study was directed toward determining Δ^9^-THC content of cannabis samples available in different regions of the United States over the last 10 years. We have divided the country into six major regions as shown in Table 1. Samples received from the states within these regions were analyzed for Δ^9^-THC content for each of the 10 years of the study. The average Δ^9^-THC content ±SD for each year (2013–2022) and for each region is shown in Figures 1–6.

**Table 1:** List of the different USA regions and the states of every region.

Regions	States												
West Regions	WA	OR	CA	AZ	NV	ID	MT	UT	WY	CO	NM		
Midwest Regions	WI	MN	MI	IN	KS	SD	ND	IL	MO	NE	OH	IA	KY
South Regions	OK	TX	AR	TN	MS	AL	GA	FL	LA				
Northeast Regions	ME	VT	NY	PA	NH	MA	RI	CT	NJ	DE	MD		
Southeast Regions	VA	NC	SC										
Alaska & Hawaii	AK	HI											

Table 2 shows the average Δ^9^-THC content of all samples seized in all regions as well as the average content of all the major cannabinoids (CBD, CBC, CBG, CBN, THCV, and Δ^8^-THC). Among the different regions, the average Δ^9^-THC fell within a narrow range (approx. 11–15%) with all other cannabinoids having <1% in all regions. The fact that all samples were of the Δ^9^-THC chemotype (drug type), the CBD content was very low (average 0.12–0.56%). For the same reason, the CBN content was relatively high (0.65–0.90%), since CBN is the final oxidation product of Δ^9^-THC (19). On the other hand, CBG content was higher in all samples than that of CBD, given that CBG is the first biosynthetic product leading to other major cannabinoids. THCV, the C3 homolog of Δ^9^-THC is usually analyzed at approximately 1% of the Δ^9^-THC content and ranges in this study from 0.07 to 0.19% in the different regions. Table 2 also lists the number of samples from each region, analyzed in this study over the 10 years period. The highest number of samples came from the western region followed by the southern region, northern region, and the midwestern region. The lowest number of samples were received from Alaska and Hawaii.

**Table 2:** Average cannabinoids content of cannabis samples from different regions of the United States for last 10 years (2013–2022).

Region	Total number of samples		THCV	CBD	CBC	Δ^8^-THC	Δ^9^-THC	CBG	CBN
West Region	2,504		0.13	0.22	0.26	0.10	13.67	0.52	0.71
Midwestern Region	1,246		0.09	0.56	0.27	0.11	12.00	0.47	0.75
South Region	2,573		0.08	0.43	0.26	0.10	12.71	0.47	0.66
Northeast Region	1,363		0.08	0.16	0.28	0.11	13.54	0.47	0.67
South East Region	569		0.07	0.25	0.27	0.10	10.82	0.41	0.65
Alaska & Hawaii	45		0.19	0.12	0.37	0.08	15.13	0.59	0.90
USA (All States)	8,300	0	0.11	0.29	0.29	0.10	12.98	0.49	0.72

While Table 2 shows data for samples from the different regions over the 10-year period, Tables 3–8 show the average Δ^9^-THC content for each year for each state, and the average for the region with standard deviation. These averages and standard deviations for the different regions are depicted in Figures 1–6.

**Table 3:** Western Region average Δ^9^-THC content for samples from each state each year, with the overall average and standard deviation for each year.

Year	WA	OR	CA	AZ	NV	ID	MT	UT	WY	CO	NM	Total number of samples	Mean	SD	% CV
2022	13.24	13.47	10.74	16.46	3.76	None	None	None	None	None	None	77	13.34	4.80	36
2021	14.75	9.61	11.1	14.7	9.58	22.99	None	None	None	None	None	110	11.50	5.08	44
2020	15.14	9.22	21.68	13.69	18.4	10.94	14.25	None	None	None	None	179	10.29	4.24	41
2019	13.29	12.47	13.09	11.13	19.21	22.32	21.65	19.62	12.87	7.22	None	141	13.36	5.04	38
2018	14.49	14.77	15.23	15.21	22.42	15.24	11.13	4.85	None	None	None	237	14.14	3.88	27
2017	16.75	11.8	12.06	10.12	20.63	22.6	10.78	13.46	11.33	None	None	264	12.43	4.55	37
2016	16.38	5.01	10.33	9.28	14.37	12.53	5.13	None	None	None	None	271	10.40	4.36	42
2015	7.48	7.91	10.94	9.93	12.87	9.42	16.14	22.35	10.15	9.03	None	321	10.43	4.53	43
2014	17.47	11.22	11.32	8.62	18.14	20.49	7.7	19.48	16.89	10.86	None	378	11.53	4.74	41
2013	15.52	8.68	10.23	9.08	14.88	14.63	4.91	9.38	17.03	12.05	10.09	498	10.62	3.66	34

**Table 4:** Midwestern Region average Δ^9^-THC content for samples from each state each year, with the overall average and standard deviation for each year.

Year	WI	MN	MI	IN	KS	SD	ND	IL	MO	NE	OH	IA	KY	Total number of samples	Mean	SD	%CV
2022	12.29	None	None	5.68	13.65	None	None	16.64	20.52	12.53	9.93	None	18.88	119	7.28	4.84	66
2021	None	None	12.58	7.9	13.84	None	15.27	13.63	10.65	None	17.16	12.9	16.44	56	13.45	2.87	21
2020	10.35	None	13.63	8.87	9.18	None	None	14.38	11.97	None	34.28	0.67	13.3	100	12.69	8.99	71
2019	2.02	None	13.11	13.53	14.25	None	None	10.15	14.26	None	11.51	15.28	4.43	89	11.05	4.68	42
2018	11.12	None	13.55	15.05	24.53	None	15.72	16.68	15.55	18.66	17.32	None	11.46	88	15.24	3.85	25
2017	None	22.82	17.82	14.58	7.04	None	None	16.62	14.36	7.75	14.83	21.73	None	84	15.61	5.39	35
2016	13.19	None	12.71	11.4	None	None	None	15.83	9.2	None	23.78	16.4	None	100	12.21	6.44	53
2015	13.32	18.16	12.5	7.29	7.35	None	None	12.32	12.36	None	12.07	17.5	10.28	97	11.30	3.59	32
2014	25.66	16.14	15.21	7.44	7.09	6.85	None	10.3	10.27	17.13	6.87	11.1	8.99	159	10.19	5.67	56
2013	21.49	9.66	11.37	6.87	14.99	None	11.48	8	10.77	16.8	9.72	13.74	8.2	278	10.41	4.21	40

**Table 5:** Southern Region average Δ^9^-THC content for samples from each state each year, with the overall average and standard deviation for each year.

Year	OK	TX	AR	TN	MS	AL	GA	FL	LA	Total number of samples	Mean	SD	%CV
2022	2.5	13.88	19.58	15.64	17.24	15.72	6.56	13.2	None	143	12.69	5.71	45
2021	None	12.74	15.66	15.8	19.38	16.45	6.83	15.14	11.31	196	12.52	3.82	31
2020	19.31	9.58	6.75	14.14	14.07	13.94	8.08	13.64	14.62	294	11.52	3.88	34
2019	14.04	10.23	14.69	6.7	15.53	13.45	8.72	15.89	8.54	223	11.71	3.45	29
2018	22.12	13.51	18.25	13.18	11.51	14.97	15.88	14.83	14.83	259	14.19	3.12	22
2017	None	10.66	19.2	17.44	29.51	16.95	17.17	14.81	12.59	243	15.13	5.67	38
2016	None	9.91	12.08	13.43	6.71	10.74	14.9	13.32	13.4	254	11.72	2.61	22
2015	5.53	8.31	17.82	10.74	14.84	20.25	11.69	12.9	14.23	443	9.68	4.55	47
2014	2.4	6.17	None	10.57	8.66	8.11	12.33	12.13	4.55	256	10.38	3.57	34
2013	10.04	8.55	None	12.71	8.36	7.86	12.12	11.96	None	247	11.46	2.03	18

**Table 6:** Northeastern Region average Δ^9^-THC content for samples from each state each year, with the overall average and standard deviation for each year.

Year	ME	VT	NY	PA	NH	MA	RI	CT	NJ	DE	MD	Number of samples	Mean	SD	%CV
2022	None	None	18.26	7.92	None	15.62	None	None	19.27	None	17.04	124	16.40	4.52	28
2021	None	17.79	15.91	3.34	15	19.7	None	12.34	18.27	16.88	16.87	108	12.20	4.89	40
2020	12.77	18	14.58	8.08	17.47	16.26	10.2	None	15.61	None	15.52	207	13.32	3.33	25
2019	16.91	None	16.49	7.42	None	17.4	14.15	19.79	12.39	11.97	13.13	81	14.45	3.69	26
2018	16.89	12.5	17.02	9.14	19.79	14.44	7.14	14.88	18.04	None	None	128	15.10	4.18	28
2017	None	20.03	18.42	11.52	None	15.33	17.93	16.76	16.76	4.86	15.99	135	15.06	4.57	30
2016	18.4	14.1	13.73	6.94	None	8.52	None	17.04	None	1.31	16.71	70	13.26	5.95	45
2015	None	None	16.22	13.17	16.01	9.38	13.63	17.08	19.03	7.53	2.41	96	12.36	5.33	43
2014	None	8.89	15.65	11.25	None	15.6	19.11	10.16	11.11	12.51	13.29	151	13.52	3.22	24
2013	12.59	10.5	14.56	8.47	1.26	12.78	4.53	17.87	10.26	7.78	9.73	231	12.64	4.59	36

**Table 7:** Southeastern Region average Δ^9^-THC content for samples from each state each year, with the overall average and standard deviation for each year.

Year	VA	NC	SC	Total number of samples	Mean	SD	%CV
2022	10.44	17.1	13	77	11.03	3.36	11
2021	13.42	16.54	None	67	14.35	2.21	14
2020	6.87	14.43	None	42	9.39	5.35	9
2019	4.89	12.78	None	66	6.56	5.58	7
2018	15.49	10.86	12.95	40	14.12	2.32	14
2017	16.73	14.08	17.14	38	16.07	1.66	16
2016	8.78	10.26	13.34	69	10.20	2.33	10
2015	14.44	10.34	9.72	39	11.73	2.56	12
2014	15.37	13.47	12.69	45	13.97	1.38	14
2013	7.95	10.16	5.25	58	8.23	2.46	8

**Table 8:** Alaska and Hawaii region average Δ^9^-THC content for samples from each state each year, with the overall average and standard deviation for each year.

Year	AK	HI	Total number of samples	Mean	SD	% CV
2022	None	None	0	0	0	0
2021	None	None	0	0	0	0
2020	None	None	0	0	0	0
2019	14.41	20.01	3	18.14	3.96	22
2018	None	20.93	4	20.93	10.95	52
2017	None	None	0	0	0	0
2016	None	16.67	3	16.67	2.49	15
2015	10.04	13.2	7	12.30	2.23	18
2014	10.31	13.62	6	12.52	2.34	19
2013	15.89	16.47	22	16.29	0.41	3

It is evident, and as expected, there is a high standard deviation among samples from the different states for each region and among the regions leading to high coefficient of variance. Over the 10-year period the coefficient of variance ranged from 27 to 44% for the Western region, 21–71% for the midwestern region, 18–47% for the southern region, 24–40% for the northeastern region, 7–16% for the southeastern region, and 3–52% for the Alaska and Hawaii region. In all cases, however, the Δ^9^-THC content was high and individual state samples had as much as 34% Δ^9^-THC (Ohio).

While these data in Tables 3–8 and the aggregate data of Table 2 deal with confiscated (illicit) samples, we have also examined the cannabinoids profile of dispensaries’ samples collected from three states with approved medical cannabis programs. Table 9 shows the average cannabinoids content of samples in dispensaries from California (San Diego and Central Valley areas), Oregon and Colorado. Comparing the data from Table 9 with those from Table 2 shows that overall, the chemical profile (cannabinoids content) of samples from any of the USA geographical regions is very comparable and follow the same pattern (profile) as the cannabinoids profile of dispensary samples from all states from which samples were analyzed.

**Table 9:** Average cannabinoids content of cannabis flower samples acquired from dispensaries in three different States.

State	Samples	THCV	CBD	CBC	Δ^8^-THC	Δ^9^-THC	CBG	CBN
Colorado	23	0.11	0.05	0.30	0.22	21.22 (13.14–30.32)	0.60	0.28
Oregon	16	0.14	0.06	0.35	0.25	24.55 (15.37–36.55)	0.97	0.38
San Diego California	47	0.13	0.05	0.30	0.17	20.90 (12.95–28.96)	0.71	0.37
Central Valley California	21	0.16	0.06	0.29	0.26	23.51 (17.32–34.66)	0.73	0.41

Δ^9^-THC ranges are shown in parentheses.

Therefore, it appears that the cannabinoids profile of all high Δ^9^-THC cannabis samples, regardless of the state or region from which the samples are seized or the state from which the sample is produced under a state medical marijuana program, is basically the same with the major cannabinoid being Δ^9^-THC (>10% for most samples) and all other cannabinoids with less than 0.5%, with the exception of CBG (<1%) and CBN (<1%).

For clinical investigations using cannabis biomass, we have also examined the chemical profile of cannabinoids of the Δ^9^-THC dominant chemovar produced for the National Institute on Drug Abuse (NIDA), Drug Supply Program (DSP). Table 10 shows the cannabinoids profile of different chemovars produced for the DSP program. Comparison of the THC content of cannabis from different regions of the USA, coupled with the observation of the other cannabinoids content shows that the chemical profile (cannabinoids-wise, at least) is very comparable. Therefore, for conducting clinical investigations using drug type cannabis (high THC Chemovars), from any part of the country, including cannabis from the NIDA Drug Supply Program, should provide similar outcomes. It is to be mentioned that the CBN content of the samples in Table 10 is much lower than those in Tables 2, 9 because samples in Table 10 are much more recent (production wise) than those in Tables 2, 9, as a result of the degradation of Δ^9^-THC into CBN over time.

**Table 10:** Chemical profiles of Δ^9^-THC dominant cannabis chemotypes produced at the University of Mississippi for the NIDA-DSP.

Variety code	Cannabinoids profile					
	THC (%)	CBD (%)	CBG (%)	CBC (%)	CBN (%)	THCV (%)
V-2	20.51	0.07	0.20	0.35	0.08	0.05
V-3	27.65	0.06	0.96	0.29	0.04	0.07
V-6	15.62	0.03	0.15	0.17	0.06	0.03
V-22	12.43	0.04	0.13	0.51	0.27	0.11
V-23	20.38	0.04	0.53	0.20	0.03	0.12
V-24	15.71	0.03	0.22	0.23	0.04	0.11
MX variety currently in NDSP	9.39	0.02	0.26	0.39	0.08	0.09

Note the similarity between the profile of these varieties (chemovars) and those found in dispensaries and on the illicit market (confiscated samples). NDSP, NIDA Drug Supply Program.

## Conclusion

This study reports on the chemical profile of cannabis plant material in use by the American public in different parts of the country, in an effort to determine differences (if any) in the types of materials in use in different regions. Cannabis samples seized in states of six geographic regions of the USA in each of the last 10 years (2013–2022) were analyzed for Δ^9^-THC content along other major cannabinoids and the cannabinoid profiles were compared.

The data presented showed that the following conclusions can be made:

1. In every region, the predominant cannabis used is that of the high THC chemotype.
2. The THC content in all regions averaged >10% and reaching over 20% in some cases.
3. The overall chemical profile of the cannabis from different regions is very similar.
4. The chemical profile of the cannabis in the illicit market (confiscated) in all regions of the country is very similar to the chemical profile of cannabis available in dispensaries operating in medical cannabis states.
5. The chemical profile of the illicit cannabis in the different regions of the USA as well as the “state legal cannabis” available in dispensaries is very similar to the chemical profile of the research cannabis available in the Drug Supply Program (DSP), provided by the National Institute on Drug Abuse (NIDA) for research in this country.

Therefore, it appears that drug type cannabis available in the USA is of closely related genetic origin and would likely have similar biological effects regardless of its geographical origin.

## Data Availability

The original contributions presented in the study are included in the article/supplementary material, further inquiries can be directed to the corresponding author.
